# Treatment of Facial Erysipelas at Camp Cody, N. M.

**Published:** 1919-01

**Authors:** 


### MEDICINE

***Treatment of Facial Erysipelas at Camp Cody, N. M.*** By ANTHONY A. AVATA and ROLLIN T. WOODYATT. *Journal A. M. A.*, Sept. 14, 1918.

Most cases of erysipelas began on the face. The disease followed drainage of the mastoid in 7 cases, abscess of the scalp in 3 cases, demonstrable ulcer in the nose in 3 cases, hordeolum in 1 case, and burns of the face in 1 case. The remaining 90 cases were called idiopathic.

Lieutenant Avata carried out a systematic employment of the method of collodion circumscription. The results were striking. All patients treated by this method averaged 3.5 days of fever and 15 days of hospital.

Collodion is painted to form a stripe half an inch wide and from half an inch to an inch in advance of the line of induration. When enough is used to produce a continuous and sufficiently deep linear constriction of the skin, the erysipelatous induration advances to the collodion but not beyond it. Within the circumscribed area the inflamed skin swells intensely, at first appearances more intensely than in non-circumscribed cases, but this is mainly an appearance due to contrast between the swollen area and the line of depression caused by the collodion.

The collodion treatment is supplemented by the continuous application of cold compresses, wet with a saturated solution of magnesium sulphate, and by general measures such as are rational in any febrile condition.

This method of treating erysipelas greatly reduces the time spent in hospital, shortens the length and height of the fever curse, lessens the toxic symptoms generally, and in facial erysipelas reduces the frequency of abscess formation to almost nothing.

